# The art of designed coiled-coils for the regulation of mammalian cells

**DOI:** 10.1016/j.chembiol.2024.06.001

**Published:** 2024-08-15

**Authors:** Tjaša Plaper, Erik Rihtar, Taja Železnik Ramuta, Vida Forstnerič, Vid Jazbec, Filip Ivanovski, Mojca Benčina, Roman Jerala

**Affiliations:** 1 Department of Synthetic Biology and Immunology, National Institute of Chemistry, Hajdrihova 19, 1000 Ljubljana, Slovenia; 2 Centre for Technologies of Gene and Cell Therapy, Hajdrihova 19, 1000 Ljubljana, Slovenia

## Abstract

Synthetic biology aims to engineer complex biological systems using modular elements, with coiled-coil (CC) dimer-forming modules are emerging as highly useful building blocks in the regulation of protein assemblies and biological processes. Those small modules facilitate highly specific and orthogonal protein-protein interactions, offering versatility for the regulation of diverse biological functions. Additionally, their design rules enable precise control and tunability over these interactions, which are crucial for specific applications. Recent advancements showcase their potential for use in innovative therapeutic interventions and biomedical applications. In this review, we discuss the potential of CCs, exploring their diverse applications in mammalian cells, such as synthetic biological circuit design, transcriptional and allosteric regulation, cellular assemblies, chimeric antigen receptor (CAR) T cell regulation, and genome editing and their role in advancing the understanding and regulation of cellular processes.

## Graphical abstract

**Figure:**
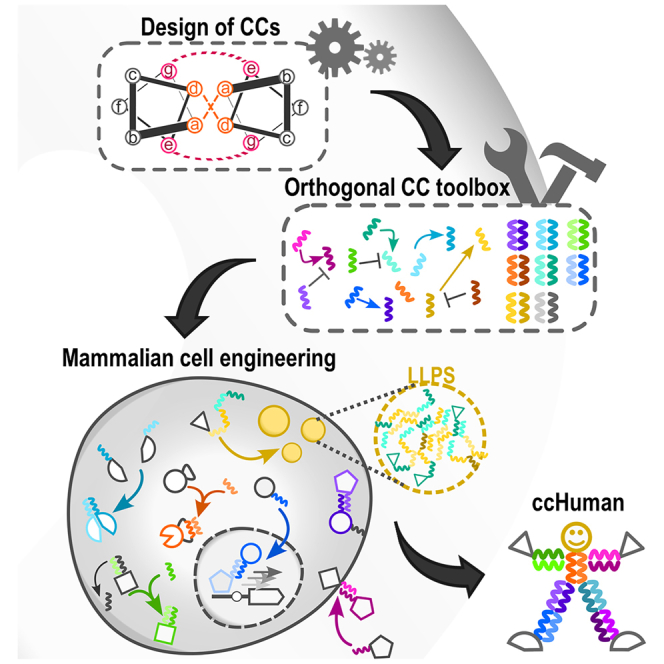

## Teaser

In this review, Plaper, Rihtar et al. highlight the modular design of coiled-coil (CC) building blocks and explore their potential for fine-tuning protein interactions and biological processes in mammalian cells. These tools hold promise for advancing bioengineering and biomedical research through applications in synthetic biology and therapeutic development.

## Introduction

Synthetic biology leverages engineering principles to design biological systems that do not exist in nature, offering exciting possibilities for medicine, biotechnology, and fundamental research.^1^ To efficiently design these synthetic systems, identifying and developing functional, modular genetic building blocks is crucial.^2^ These interchangeable parts, similar to Lego bricks, can then be assembled into complex biological systems with novel functionalities. One approach to discovering these parts involves identifying the modules used in natural evolution, particularly those found in endogenous genetic regulatory and signaling pathways,^2^^,^^3^ where modules such as coiled coils (CCs) have been used in diverse molecular machines.^4^

The regulation of mammalian cells can be achieved at various levels, including DNA, RNA, or proteins. Synthetic transcription factors are the most established and well-characterized modular regulators to date.^5^ These engineered proteins are composed of two interchangeable domains: a DNA-binding domain (DBD) and a transcriptional effector domain. The modularity of these proteins is exemplified in the design of DBDs, based on zinc fingers, transcription activator-like effectors (TALEs), or CRISPR dCas9/gRNA-based targeting for sequence-specific DNA binding.^5^^,^^6^^,^^7^^,^^8^ By customizing DBDs and combining them with various transcriptional effector domains, diverse gene outcomes can be achieved, either by activation or repression of transcription. Moreover, combining synthetic transcription factors allows for constructing modular transcriptional devices that process multiple inputs and generate the appropriate outputs according to the underlying logic. This approach has successfully yielded transcriptional logic gates in mammalian cells, integrating multiple signals to produce the desired response.^9^^,^^10^^,^^11^

Similarly, mammalian signal pathways can be reprogrammed and repurposed using natural modular signaling protein domains like scaffolds/adapters for tyrosine kinases, proteases, and transmembrane receptors.^12^ These proteins can be rewired to respond to selected stimuli and produce novel signaling responses, as exemplified by engineered receptor platforms like chimeric antigen receptors (CARs), Notch receptors,^13^^,^^14^ and several other designs as reviewed by Manhas et al.^13^ These platforms incorporate modular extracellular sensor domains (scFvs, nanobodies, or other protein domains) for recognition of user-defined ligands and subsequent signal transduction within cells. Additionally, sensor protein domains responsive to external stimuli (small molecules, light, and ultrasound) can be fused to proteins of interest, enabling signaling with precise temporal and spatial control.^15^^,^^16^ Alternatively, light and ligand-sensing domains can also be inserted into protein loops to introduce designed allosteric regulation, although this requires more optimization.^17^

Repurposing natural protein-derived modules offers a convenient starting point for building regulatory switches, but it can sometimes lead to unintended interactions with endogenous cellular processes resulting in unexpected outcomes. To achieve more orthogonal synthetic protein devices, synthetic biology is increasingly embracing the emerging field of *de novo* protein design, which enables the engineering of entirely new modular protein structures.^18^^,^^19^ Nature has provided a blueprint of interacting domains through naturally occurring CC motifs, such as those found in the leucine zipper domains of transcription factors. These motifs facilitate specific and controlled interactions between protein domains, crucial for regulating cellular processes. By leveraging well-understood rules governing protein-protein interactions, researchers can engineer orthogonal-designed CC peptide pairs with tailored properties. Strategic design and incorporation of these CCs into target proteins allow for influencing cellular pathways like signal transduction or gene expression but also cellular localization. This review highlights the growing potential of *de novo* interacting protein domain design, focusing on the use of designed CC modular building blocks as a powerful tool for precise control of protein-protein interactions and regulation of protein function, with an emphasis on mammalian cells.

### CC peptide interaction toolkit for synthetic biology

Recent advances in machine learning-based protein structure prediction^20^ and generative protein design^21^^,^^22^^,^^23^^,^^24^ opened tremendous potentials for engineering biological systems, by generation of new scaffolds and protein binders that enable the design of new protein functions. However, smaller protein modules designed for multiple, specific, and orthogonal interactions can be equally powerful for the regulation of biological processes.

Inspiration for modular protein design came from relatively straightforward miniproteins such as zinc fingers and leucine zippers, with clear and robust sequence-to-structure relationships. Their robust versatility and small size are the reason that these modules are present in hundreds of proteins within the proteome of mammalian cells.^25^^,^^26^ A rational approach to protein design and engineering has been particularly fruitful in the development and understanding of special classes of helical bundles and CCs.^27^^,^^28^

**Figure 1:**
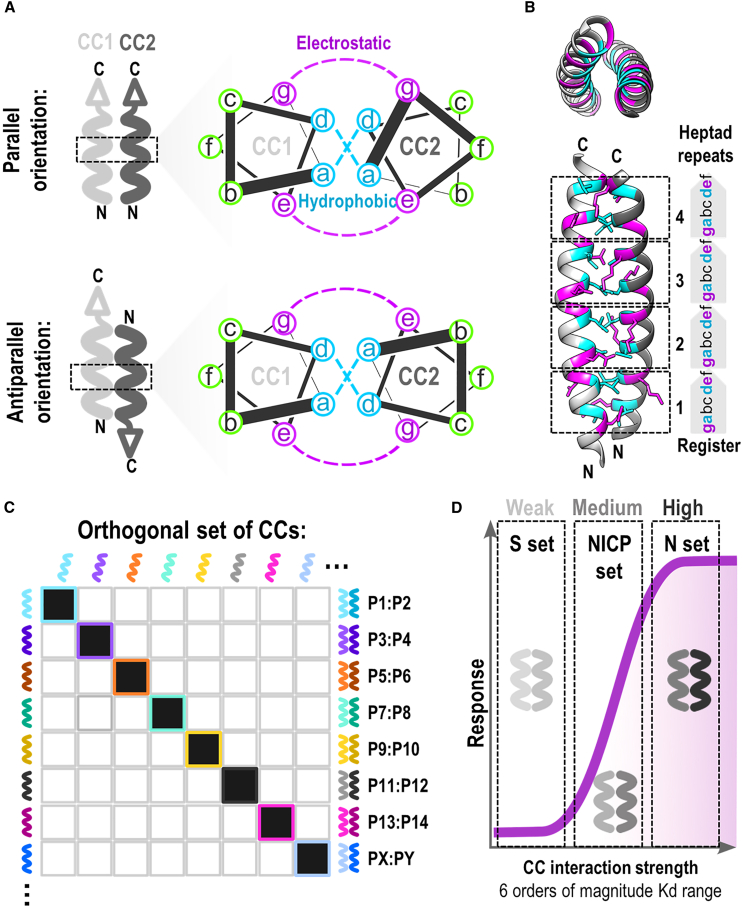
Design of coiled-coil (CC) peptides (A) Schematic and helical wheel representation of parallel (top) and antiparallel (bottom) CC heterodimers. Positions of amino acid residues in heptad repeats are labeled (*a*,*b*,*c*,*d*,*e*,*f*,*g*). Hydrophobic (cyan) and electrostatic (magenta) interactions between residues are shown in dashed lnes. (B) Structure of a parallel CC heterodimer. Residues involved in electrostatic and hydrophobic interactions are colored magenta and cyan, respectively. Heptad repeat residues are shown. (C) Representative matrix of a designed set of CC heterodimers. Dark squares indicate the expected interaction between designed heterodimers. (D) Schematic representation of different sets of designed CC peptides (S, NICP, and N) that differ in interaction affinities and thermal stabilities (weak to high).

CC peptides form a structural motif found in numerous natural proteins, playing roles in diverse biological processes.^4^^,^^29^ These peptides are characterized by their helical structure, typically composed of two or more alpha helices winding around each other, either in parallel or antiparallel orientation (Figures 1A and 1B).^30^^,^^31^^,^^32^ One distinctive feature of CC peptides is their pattern of amino acid residues. The most frequent canonical CCs comprise heptad repeats that contribute to the amphipathic nature of CC peptides, with alternating hydrophobic and polar residues driving the assembly. This repeat is denoted *a-b-c-d-e-f-g* in one helix and *a′-b′-c′-d′-e′-f′-g′* in the other,^29^ with geometry already postulated by Francis Crick.^33^ The positions of specific amino acid types within the heptad repeat define the stability and pairwise interactions of CCs. The hydrophobic residues can typically be found at the *a* and *d* positions and they form a hydrophobic core. In addition to hydrophobic, electrostatic interactions play a key role in CC stabilization and selectivity. Contributing to these interactions are positions *e* and *g* where polar or charged amino acid residues are usually positioned. The remaining *b*, *c*, and *f* positions are, like *e* and *g*, solvent-exposed and can affect the helical propensity of a CC. The pattern of electrostatic and hydrophobic interactions defines the overall stability as well as the orientation of the CC structure.^34^ Deviations, such as stammers and stutters, can influence the oligomeric state and helix orientation. Additionally, the interface residues further affect oligomeric assembly, with specific combinations favoring dimers, tetramers, or higher-order oligomers. Moreover, variations in helix packing, such as bifaceted interfaces, contribute to structural diversity, yielding intricate assemblies ranging from helical bundles to open sheets further expanding possibilities of CCs utility.^35^^,^^36^ Four helical bundles (4HB) can take the integration of an input signal even further by coupling three or four signals to an output as demonstrated by Merljak et al.^37^ By implementing single components of four helical bundles to split proteins, we can design multi-component transcription factors or integrate signals from two CARs to enable downstream signaling in the case of dualCAR-T-4HB. 4HBs have been designed based on the hydrogen bonding network to increase the specificity in comparison to hydrophobic interactions, which enabled the generation of orthogonal combinations of split segments,^38^^,^^39^ although larger interaction interface in comparison to CC dimers tends to have larger nonspecific interactions.^40^

The design of CC interacting peptides therefore follows well-defined rules.^29^^,^^41^^,^^42^^,^^43^ Understanding these rules has paved the way for the development of a toolkit for synthetic biology. Several tools and algorithms for CC design (e.g., iCipa) enabled us to design *de novo* CC sets of orthogonal pairs with tunable stability, which may affect biological properties.^44^^,^^45^ We can tailor CC dimers as well as higher oligomers for specific applications, as reviewed in detail by Woolfson et al.^32^ Protein-protein interaction specificity is commonly achieved through backbone shape complementarity; however, it can be challenging to generalize. CC heterodimers have limited design options due to the restricted geometry of interactions across the interface. Heterodimeric interaction specificity can be further enhanced using designed buried hydrogen bond networks rather than relying predominantly on hydrophobic interactions as shown by Chen et al., using Rosetta-based design.^39^ Achieving orthogonality involves designing CC pairs that do not cross-interact with other cellular components (Figure 1C). In addition to natural CCs, which are still mainly used as homodimers, CC pairs have also been designed. Since the initial single designed pair, combining all-acidic (E) and all-cationic (K) pair,^46^^,^^47^ researchers expanded the number of orthogonal pairs (“peptide velcro”) that have been demonstrated to work well in many applications. Heterodimerizing leucine zipper peptides have been designed with a range of pIs and stabilities in the femto- to nanomolar range through altering residues at *a*, *d*, *g*, and *e* positions.^48^ While CC stability increases in regard to peptide chain length, inclusion of polar residues within the hydrophobic core allows for fine-tuning of stabilities as shown by Woolfson et al., who engineered a set of parallel heterodimeric CCs with variable lengths and tuned stabilities due to incorporation of Asn residues at the site of a central heptad.^49^ Based on synthetic peptides to interact with natural leucine zippers, a set of CCs was prepared, and their interaction matrix was experimentally determined.^50^^,^^51^^,^^52^ In addition to the length of CCs,^53^ the local helical propensity of interacting peptides and intramolecular salt bridges have been used to tune the stabilities of CC dimers over a wide range.^34^ This approach was successfully used to modulate the stabilities in an orthogonal set of CCs without affecting their binding preferences. Previously, four parallel and orthogonal peptide pairs composed of four heptad repeats have been designed. The design was achieved by combinations of a pattern at *e*/*g* and *a* positions, while also implementing the negative design motif based on the burial of asparagine residues.^54^ This designed set of CCs was further expanded by the design of two additional sets of CCs (termed NICP set),^55^ and by changing noninteracting amino acid residues within CCs to influence the stability and binding affinity of a dimer and generating the strongest four-heptad CC pairs with low nanomolar affinity^56^ (Figure 1D). The highly stable N-set was used for reporter reconstitution and detection of the SARS-CoV2 virus S protein-mediated cell fusion. Designed CC pairs were further massively screened for the pairwise orthogonality using RNA sequencing in bacterial cells to generate the largest set of orthogonal CCs, comprising more than 20 pairs, combining homodimers and heterodimers and additionally provided information to refine the energy function to predict the stability of CCs.^45^

On the other edge of the stability spectrum, destabilized CCs generated the weakest interaction set of CCs reported so far (S set), with affinities in the millimolar range. They were used for the generation of liquid-liquid phase separated (LLPS) condensates, membrane-less compartments involved in numerous biological processes, from gene regulation, signal transduction, and stress response in mammalian cells.^57^ These weakly interacting CCs fused to generate multivalent polypeptide chains that were required to form liquid condensates in mammalian cells (CC-LLPS). The stability of CC pairs, their number, linkers, and sequential arrangement governed the transition between diffuse, liquid, and immobile condensates. Similar to this approach, multivalent CC sequences with precisely tuned weak interactions were designed to generate liquid protein condensates.^58^ These engineered CCs formed dynamic droplets within *Escherichia coli* under the physiological conditions, serving as functional compartments that enabled the colocalization of enzymes within the cell, potentially enhancing their activity. In contrast to approaches utilizing destabilized CCs, Zhang et al. described an approach using *de novo* designed homo-oligomeric CCs (HO-Tag) to introduce multivalency of protein-protein interactions, driving phase separation.^59^ This method, termed SPARK (separation of phases-based activity reporter of kinase), was employed to visualize kinase activity in mammalian cells. SPARK utilizes GFP-tagged reporter molecules that undergo phase separation upon kinase activation. The resulting phase separation leads to the formation of bright, fluorescent droplets, enabling easy observation of kinase signaling dynamics within living organisms.

Similar to the principles of DNA nanotechnology, the assembly of diverse CC nanostructures can be achieved by combining peptides that form CCs into longer polypeptide chains.^60^^,^^61^ This methodology relies on the concatenation of orthogonal CC-forming peptides within single or multiple polypeptide chains. These chains undergo self-assembly, guided by the specific pattern of pairwise interactions between the CC-forming modules.^60^ This approach enabled the design and fabrication of mono- and multimeric polyhedral shapes both *in vitro*^62^^,^^63^^,^^64^^,^^65^ and *in vivo*.^60^^,^^66^ Furthermore, Aupič et al. expanded the CC toolkit by designing Zn(II)-dependent CCs that can self-assemble into a multimeric CC-based bipyramidal protein cage in response to Zn(II) ions.^67^ Coiled coils have also been used to make materials,^31^ to cluster biosynthetic enzymes for metabolic engineering,^68^ and in several applications in biomedicine.^69^^,^^70^^,^^71^^,^^72^

### CCs as protein interaction modules

**Figure 2:**
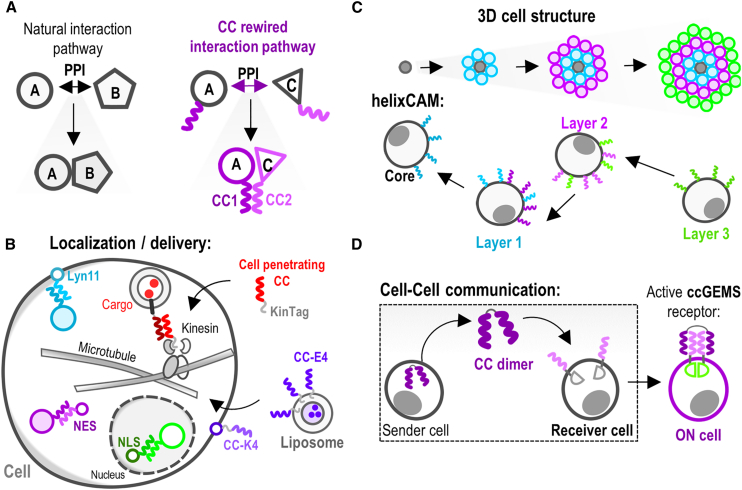
Protein interaction devices based on coiled coils (A) Two-component transmission systems based on protein-protein interactions. (B) CC-based cell compartment localization. CC dimers were used to direct subcellular protein localization to the plasma membrane, cell nucleus, or cytosol. Cargo delivery into cells and subcellular targeting were also successfully demonstrated by the implementation of cell-penetrating peptides and CC-mediated liposome fusion. (C) Building high-order cellular organizations using CC dimers. A platform for engineering synthetic cell adhesion molecules, termed helixCAM, implemented various CC dimers to transmembrane domains. This enabled the formation of multicellular aggregates of selected mammalian cells. (D) CC dimers-based intercellular communication. A small ubiquitin-like modified (SUMO) tag was fused to a CC dimer, which was secreted by mammalian cells (sender population). Upon binding to the complementary CC peptides on the receiver population a receptor was activated.

Two- or multi-component transmission systems based on protein-protein interactions govern biological processes such as signal transduction, gene expression, trafficking, defense, and many others.^73^ We can manipulate these information flows by controlling protein-protein specificity with orthogonal interaction domains (Figure 2A). Here, we showcase examples of applications of CC pairs as tools for mediating protein-protein interactions and regulation of cellular processes in mammalian cells.

#### From intracellular localization and delivery to orchestrating cellular organization and communication

CCs have diverse applications ranging from directing intracellular protein localization to facilitating cell-cell communication. CC dimers can be used to direct subcellular localization, which has been demonstrated by Lebar et al., who used the NICP set to multiplex localization of proteins to the plasma membrane, cell nucleus, or cytosol.^55^ Another study demonstrated that newly designed hetero-dimeric CCs are effective for delivery into cells and subcellular targeting.^74^ Namely, the arginine-rich basic CC peptide was used as a delivery vehicle, capable of cell penetration, while the sequence of its complementary anionic partner was fused to subcellular proteins and introduced into the cells via synthetic genes. A KinTag sequence was developed, which binds the endogenous kinesin-1 light chain and as such provides a synthetic adapter between the kinesin-1 heavy chain motors and selected cargoes (Figure 2B). Similarly, Li et al. demonstrated that the K5 CC does not only act as a cell-penetrating peptide but can also bind other proteins (e.g., GFP and Cas9) containing its partner peptide E5.^75^ Another example of using CCs for drug delivery was demonstrated by Yang et al., who used synthetic heterodimeric E/K coils to induce targeted liposome fusion mediated by the CC formation.^76^ They modified cells with lipopeptides fused to a K_4_ CC (the number 4 corresponds with the number of heptad repeats within the peptides) and upon the addition of E_4_ CC-decorated liposomes, membrane fusion occurred, resulting in cytosolic delivery of various compounds, including fluorescent dyes and cytotoxic drugs. This system can be used for *in vivo* drug delivery, as they injected E_4_ CC-decorated liposomes carrying cytotoxic drug doxorubicin into zebrafish xenografts of a HeLa cancer cell line expressing K_4_ CCs on the membrane, which resulted in diminished cancer proliferation in the xenograft.^77^ Another study demonstrated the use of E/K CCs in spatiotemporal control of liposome accumulation in cancer cells, in which light was used as an external trigger to induce membrane fusion.^78^ To further improve cell delivery, CC-modified nanoparticles (LNPs) were developed. They included a lipopeptide E_4_ to LNPs and exposed them to target cells carrying lipopeptide K_4_ that successfully internalized the cargo, resulting in 63-fold increase in protein expression in comparison to unmodified LNPs. Recently, the same research group developed novel dimer K_4_ CCs and used them together with E_4_-modified liposomes to enhance liposomal drug delivery.^79^ While these studies show promising results in terms of improving cell delivery, target cells also need to be modified to express the lipopeptide, which may limit applications.

CCs can also be used to direct localization in bacterial cells. To guide selected proteins to a specific intracellular position in bacterial cells, Lee et al. developed a cytoscaffold composed of a bacterial microcompartment shell protein and two complementary CCs (acidic and basic peptides), which resulted in an improved metabolic efficiency through enzyme colocation.^80^

Cellular assemblies can be built using CC dimers. One example is helixCAM, a platform for engineering synthetic cell adhesion molecules, where various CC dimers were fused to transmembrane domains, which enabled the formation of multicellular aggregates of selected mammalian cells, transforming suspension cells to adherent cells and facilitated specific spatial patterning (Figure 2C).^81^ Furthermore, CC dimers can also be used to regulate intercellular communication. Makri Pistikou et al. developed an orthogonal platform for synthetic communication in mammalian cells.^82^ A small ubiquitin-like modified (SUMO) tag-fused CC dimer was designed, which was secreted by mammalian cells (sender population). Connected with the receiver population carrying complementary CC peptides, CC binding resulted in receptor activation (Figure 2D). Additionally, they demonstrated the synthetic receptor-expressing cells can perform two-input logic (AND and OR gate) operations.

#### CC dimers for tuning gene expression

**Figure 3:**
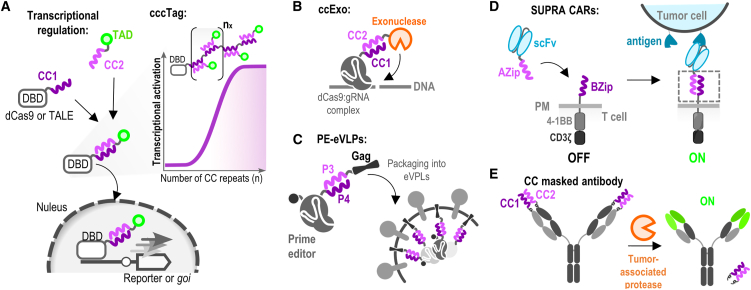
Coiled-coil-based engineering of transcriptional regulation and modification of antibodies (A) Split synthetic transcription factor-based system composed of DNA-binding domains (TALE or dCas9) and effector domains, both fused to CC pairs. CC peptides enable the reconstitution of DNA binding and activation domain into an active transcription factor. (B) Recruitment of an exonuclease to the Cas9/gRNA complex via CC interaction for increased gene editing efficiency. (C) Prime edit (PE) facilitated by CC. Virus-like particles (VLPs) were used to deliver a double-stranded break-free gene editing system into cells. (D) SUPRA CAR system, composed of a zipCAR and zipFv, for targeting tumor cells. Fused to each of the two components, CC (leucine zipper) served for reconstitution into a functional receptor. (E) Engineered antibodies that can be selectively activated in the tumor microenvironment. Heterodimeric CC domains served as a steric hindrance on complementarity-determining regions that were exposed only upon action of tumor-associated proteases.

Controlling gene expression is crucial in engineering biological systems as well as for gene therapy.^83^^,^^84^ In this context, CCs can recruit transcriptional effector domains (activators or repressors) to specific promoters, enabling fine-tuned gene regulation. Tanenbaum et al. designed SunTag, a protein scaffold composed of repeating CCs (GCN4) that was fused to dCas9.^85^ Multiple copies of antibody-gene regulatory effector domains (VP64 activation domains) fusion were recruited to dCas9 via antibody-CC interaction, which enabled amplified transcriptional activation of selected endogenous genes. Lebar et al. used the designed NICP set to enhance the regulation of gene transcription. This was achieved by utilizing a split synthetic transcriptional factor-based system composed of DNA-binding domains (TALE or dCas9) and effector domains, each fused to a peptide of the CC pairs (Figure 3A).^55^ This facilitated the recruitment of the effector domain to the promoter. Moreover, a concatenated CC peptide tag (CCC-tag), comprising multiple copies of a coil peptide, enabled tunable control of the stoichiometry of the bound transcriptional activation domains. This was used to develop powerful CRISPR-dCas9-based transcriptional activators, the activity of which was demonstrated *in vitro* and *in vivo*. In comparison to SunTag, the CCC-tag system has a smaller genetic footprint, which is advantageous when considering therapeutic applications, and additionally, it enables the recruitment of multiple and different effector proteins that can modulate transcription. CC hetero- and homodimers have also been used to generate combinatorial transcription factors to introduce multistability into mammalian cells.^86^

#### CC dimers for genome engineering

Combining various effector proteins with genome editing enzymes provides an opportunity to further expand the genome editing molecular toolbox, for enhanced potency or broadening of the functional features of these systems. Non-covalent tethering of proteins via CCs can provide an advantage concerning protein folding issues of large genetic fusions and size constraints, especially in the context of viral vector size limitations for therapeutic applications. Lainšček et al. utilized CC-based recruitment of an exonuclease to the Cas9/gRNA complex that resulted in greater gene disruption efficiency (Figure 3B). Cas9 coupled to an exonuclease via a CC-forming peptide pair demonstrated not only a larger frequency of the target gene inactivation but also a larger size of deletions in comparison to Cas9 alone, co-expressed with or in a direct genetic fusion to the exonuclease.^87^ Another instance involved the delivery of the double-stranded break-free gene editing system, prime edit (PE), into cells using virus-like particles (VLPs). Here, the prime editor was fused to a retroviral Gag polyprotein, guiding the packaging of the cargo protein within the VLPs (Figure 3C). By employing a CC-based recruitment strategy to tether the prime editor to the retroviral Gag polyprotein, a notable enhancement in editing efficiency was observed compared to directly fusing PE with Gag.^88^ Conversely, when employing CC-based tethering of components in the innovative click editor, where a Cas9 nickase is combined with an ssDNA-binding HUH endonuclease and a DNA polymerase to enable double-stranded break-free genome editing, comparable efficiency was achieved as with direct fusion.^89^ This indicates that the effectiveness of CC-supported genome editing may vary depending on the context.

#### CC mediated immunotherapies

One of the most promising approaches to cancer immunotherapy is CAR T cell therapy, which shows remarkable results in the treatment of hematologic malignancies for targeting and eradication of cancer cells. Safety and efficacy concerns underline the need for further improvements where synthetic biology is playing a strong role.^90^ To enhance the specificity, safety, and programmability of CARs, a novel “split, universal, and programmable” (SUPRA) CAR system was developed.^91^ The system is composed of the universal receptor expressed on T cells (zipCAR) and a scFv adaptor molecule (zipFv) that targets tumor cells (Figure 3D). To form zipCAR, the intracellular signaling domains and a transmembrane domain were fused to a leucine zipper, which enabled the reconstitution of the two domains into a functional receptor via CC interaction. The authors demonstrated that the activity of the SUPRA CAR can be stimulated through several mechanisms, thus avoiding excessive activation, and is capable of responding to combinations of antigens. They adjusted the concentration and expression levels of zipFv, the affinity of leucine zipper (zipFv and zipCAR), and the affinity of zipFv to the target antigen. By doing so, they modulated the output level of SUPRA CAR and developed a dually inducible orthogonal SUPRA CAR system that was able to regulate different T cell subsets independently. The same group then upgraded this design by developing VIPER (versatile protease regulatable) CARs, consisting of zipCAR and zipFv, which formed ON/OFF switch circuits. In this case, zipCAR was composed of two parts: the first consisted of an extracellular leucine zipper, a transmembrane domain, a CD28 costimulatory domain, and an NS3-binding peptide; while the second consisted of a membrane-tethered component containing a DAP10 ectodomain, costimulatory 4-1BB domain, catalytically inactive NS3, and CD3ζ signaling domain. Once zipFv recognized the tumor antigen, VIPER CAR was activated and its activity was controllable with the antiviral protease inhibitor grazoprevir.^92^ To further improve the efficacy of CAR T therapy, Cordoba et al. developed a CAR T therapy termed AUTO3 with dual specificity against tumor antigens CD19 and CD22.^93^ AUTO3 represented an advancement in CAR T cell treatment, where CCs play an important role in its design. CD22 CAR functionality was significantly enhanced through the incorporation of a homopentameric CC spacer derived from cartilage oligomeric matrix protein (COMP). The AUTO3 CAR T therapy was used in the phase 1 trial in pediatric and young adult patients with relapsed refractory B cell acute lymphoblastic leukemia and a favorable safety profile was shown, with no dose-limiting toxicities, cases of treatment-related severe cytokine release syndrome or neurotoxicity reported. Notably, the one-year overall survival was 60% and the event-free survival was 32%.

A protein-based immunotherapy approach using CC dimers was also developed. Trang et al. engineered antibodies that can be selectively activated in the tumor microenvironment.^94^ They fused antibodies to heterodimeric CC domains, which sterically obstructed the complementarity-determining regions. Only upon exposure to tumor-associated proteases (e.g., matrix metalloproteinase 2 and 9), the CC dimers were cleaved and antigen binding was restored (Figure 3E).

### CCs as a tool for modular allosteric regulation of protein function

**Figure 4:**
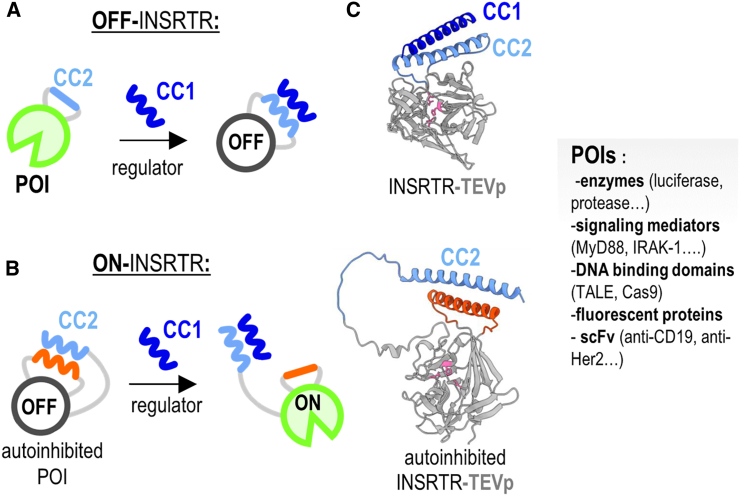
Coiled coils as allosteric modulators of protein function (A and B) The principle of INSRTR allosteric regulation is based on the insertion of a CC-forming peptide into a target site of a protein, allowing the protein to retain its function. This setup allows the design of either (A) OFF-INSRTR, where the binding of a regulatory peptide disturbs protein function or (B) ON-INSRTR, where regulatory peptide releases the autoinhibitory loop, allowing the protein to regain its function. (C) 3D structure of OFF (top) and ON (bottom) INSRTR-TEV protease. The active site is marked in magenta.

Allosteric protein regulation plays a central role in cellular processes, as a dynamic mechanism through which proteins modulate their activity in response to diverse signals. The binding of an allosteric regulator to a specific site of a target protein induces a conformational change that propagates throughout its structure. These changes can activate or inhibit the protein’s function.^95^^,^^96^ Several approaches for protein regulation via allostery have been reported; notably, introduction into individual proteins by mutations^97^ and insertion of folded protein domains^98^ or domains regulated by small molecules.^99^^,^^100^ Methods used for the regulation of protein function often involve complex mechanisms that can be challenging to design. However, recent advancements in protein engineering led to the development of innovative platforms such as the INSRTR (Insertion of a peptide to Regulate protein function) system (Figure 4),^101^ which leverages the unique properties of CCs as allosteric regulators. In this platform, the peptide is inserted into a target site of a protein, allowing the protein to retain its function while providing a site for the binding of an appropriate regulatory peptide, such as a complementary CC-forming peptide. Upon binding of a regulatory peptide, the inserted and regulatory peptide form a CC dimer, adopting a helical conformation. The formation of an alpha-helical structure expands the distance between the inserted termini in the loop and introduces dynamic constraints. This subtle perturbation disturbs the conformation at the site crucial for protein function. INSRTR enables the design for either inactivation (OFF-INSRTR) or activation (ON-INSRTR) of selected protein functions, which can be achieved through an intramolecular autoinhibitory CC dimer. The adjustable properties of designed CCs further enhance the adaptability of the platform, making it a powerful tool for precise control of protein function. Fine-tuning of CC affinities is especially beneficial for ON-INSRTR constructs where the inserted and inhibitory peptide form an intermolecular interaction. The interaction needs to be strong enough for efficient inhibition and sufficiently weak so the regulatory peptide can compete with it, which was achieved with a P7:N8 combination of CCs.^102^ The intramolecular fusion with regulatory peptides further offers the possibility to engineer complex regulatory circuits with a fast response, expanding the repertoire of applications for this platform. One important feature of designed CC dimers used in the INSRTR platform is their incorporation of negative design principles. These principles enhance the orthogonality of the CCs in mammalian cells. By using different combinations of CCs and incorporation of cleavage sites for orthogonal (split) proteases, complex levels of protein regulation can be achieved. The broad applicability of the INSRTR platform has been successfully demonstrated on ten different proteins, which include a range of unrelated proteins such as enzymes, signaling kinases and mediators, DNA-binding proteins as transcription regulators, fluorescent proteins, and the single-chain variable fragment (scFv) of antibodies. An important advantage of the INSRTR platform is its ability to achieve precise and adjustable regulation with a small genetic footprint, thus minimizing potential immunogenicity concerns and opening possibilities for the engineering of tailored regulation for a wide range of proteins.

### Split proteases and CC-based molecular circuits

**Figure 5:**
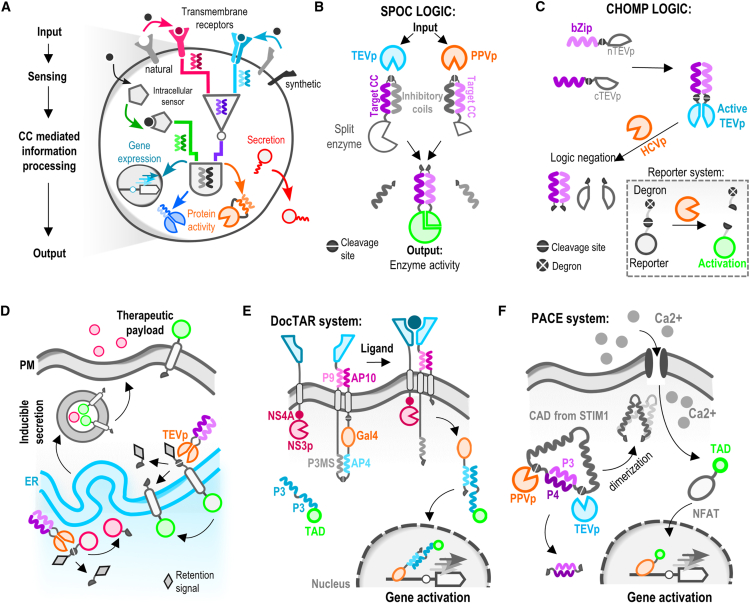
Modular protease- and coiled-coil-based molecular circuits (A) Designed molecular circuits that can sense different input signals, process information, and trigger complex cellular responses. (B) Split-protease-cleavable orthogonal-CC-based (SPOC). Strategically positioned protease cleavage sites and CCs enabled different levels of binding and competition. This allowed the creation of a range of two-input Boolean logic circuits. (C) Logic and circuits of hacked orthogonal modular proteases (CHOMP). Similar to SPOCK, CHOMP relies on protease-induced regulation; however, an additional type of regulation is added through the implementation of degrons. (D) Engineered inducible secretion of proteins from the endoplasmic reticulum (ER), lumER, RELEASE, and membER. CC-operated split protease system applied to an inducible protein secretion system enables regulated secretion of therapeutic proteins by an array of inputs. (E) CC-based two-part transmembrane synthetic receptor termed DocTAR. One part carries a protease and the other contains a protease-responsive split transcription factor fused to an autoinhibited antiparallel CC. Ligand-based bridging enables the release of a split transcription factor that is reconstituted in the cytosol by a third part of the system via CC interaction. (F) CC modified proteolytically engineered activators of calcium channels (PACE). The system is composed of a CAD protein domain, which can activate Orai Ca^2+^ channels, and a CC pair that renders the domain inactive. Activation was achieved by strategically incorporating protease cleavage sites positioned at the end of the CC inhibitory region.

One important aim of synthetic biology is the design of molecular circuits that can sense different input signals, process information, and trigger complex cellular responses (Figure 5A). CCs, with their ability to mediate interactions between engineered protein components, allow for the creation of such complex post-translational circuits in mammalian cells. Notably, when combined with orthogonal split proteases, they enable the formation of quickly responsive and highly modular systems. Two systems are employed this mode of action: split-protease-cleavable orthogonal-CC-based (SPOC) (Figure 5B)^103^ logic and circuits of hacked orthogonal modular proteases (CHOMP) (Figure 5C).^104^ Both SPOC logic and CHOMP use CC dimerization to reconstitute inactive split proteases for active enzymes. The CHOMP relies on the protease-induced regulation of the activity of degrons fused to the target proteins. SPOC on the other hand directly activates or inactivates the effector protein through the release of autoinhibitory CC interactions. These protein-protein interactions create multi-layered operations as designed proteolysis-based signaling pathways. By strategically positioning protease cleavage sites and CCs, different combinations of binding and competition can be achieved, allowing for the creation of a range of two-input Boolean logic circuits.

The combination of proteases and CCs, both in parallel and antiparallel orientation, has proven useful in engineering synthetic circuits that control endogenous processes in mammalian cells. Praznik et al. implemented some elements of SPOC logic for engineering inducible secretion or membrane translocation of proteins from the endoplasmic reticulum (ER) (Figure 5D).^105^ The pre-synthesized proteins were retained in the ER until the signal-triggered activation of proteases, which cleaved off their retention signal. They were located either in the ER lumen (in the case of lumER) or in the cytosol (in the case of membER), enabling the release of proteins from cells. Rapid release of therapeutic proteins like hormones and plasma membrane translocation of CAR receptors from the ER was successfully demonstrated.^105^ This exemplifies CC-based circuits can also operate in distinct cellular compartments enabling new therapeutic strategies. Vlahos et al. engineered a similar CC-operated split protease system and applied it to an inducible protein secretion system, termed retained endoplasmic cleavable secretion (RELEASE). This system showcases the modularity of protease circuits by implementing previously developed protease activating systems such as MESA (modular extracellular sensor architecture)^106^ and Tango^107^ to enable regulated secretion of therapeutic proteins by an expanded array of inputs.^108^

Another example of CC-based signal transduction coupled to proteases is a system termed DocTAR.^109^ Here, two antiparallel CC pairs were used to engineer highly modular and orthogonal two-part transmembrane synthetic receptors with low background signaling activity (in comparison to GEMS (generalized extracellular molecule sensor) or MESA) (Figure 5E).^13^ The system is composed of two synthetic receptor chains: one carrying a protease and the other containing a protease-responsive split transcription factor fused to an autoinhibited antiparallel CC and linked via a protease cleavage site. When the membrane-anchored split transcription factor is bridged by the ligand at the extracellular side, it is cleaved by the protease and released from the membrane. Subsequently, the split transcription factor is reconstituted in the cytosol by a third part that comprises a replacer coil fused to a transcription activation domain. To improve the ON/OFF ratio, another antiparallel CC pair was introduced into the extracellular sites of the synthetic receptor. Such multi-mode control and implementation of CC-based logic yielded low background signals of the engineered DocTAR receptors.

Autoinhibition by intramolecular CC interactions represents a powerful concept for the regulation that was used for the regulation of proteolytically engineered activators of calcium channels, known as PACE.^110^ In this study, the researchers used a STIM1 protein domain called CAD, which can activate Orai Ca^2+^ channels and an intramolecular CC pair that rendered the domain inactive (Figure 5F). The construct can be activated by proteases since the cleavage sites were introduced adjacent to the CC autoinhibitory region. This addition expanded the capability of fast-acting synthetic circuits to activate Ca^2+^-dependent processes, holding potential for applications in neuroscience and cellular signaling.

## Conclusions and outlook

CCs stand at the interface of structural biology and synthetic biology and represent a great example of applying modular combinations of protein design with potent applications for the regulation of cellular processes. The frequent occurrence of CC motifs in natural proteins underlines their significance, and the ability to engineer these peptides opens up a powerful toolkit for advancements in biotechnology and medicine. By manipulating interactions among modular-engineered CCs, scientists can design a wide array of applications. This regulatory capacity is particularly appealing in the context of mammalian cells, offering the potential for controlling and understanding biological processes through the strategic engineering of diverse processes.

CC dimers as designed interaction domains present a distinct advantage over naturally occurring dimerization domains as they avoid interference with natural components. This was demonstrated in mammalian cells by analysis of its interaction with human proteome.^55^ The well-understood design rules governing CCs allow for precise fine-tuning of affinities, stability, and orthogonality.^27^^,^^34^ This level of control is achieved by strategically positioning specific amino acid residues and adjusting the length of CCs to influence specificity. The result is the ability to design and engineer CC peptides with desired functionalities and stabilities, tailoring them for specific applications without their cross-reaction to other cellular components.

The versatility of CCs extends across a broad spectrum of applications, and the selection of specific CCs and their utility varies depending on the specific application. They have found use in transcriptional regulation,^55^^,^^85^ reporter reconstitution,^56^^,^^111^ they contribute to the regulation of liquid-liquid phase-separated condensates,^57^ they facilitate inter- and intracellular protein localization,^55^^,^^74^^,^^75^^,^^81^ and enhance cell-cell communication.^82^ The development of platforms such as helixCAM for engineering synthetic cell adhesion molecules and enabling synthetic communication in mammalian cells further exemplifies their utility.^81^ By using CCs, we are also able to improve regulation of gene expression and genome engineering. Notably, this was achieved through the non-covalent coupling of Cas9 to an exonuclease via a CC-forming peptide.^87^ It was also shown that CCs can modulate the safety and efficacy of CAR receptors.^91^^,^^92^ Furthermore, CCs can be used in allosteric protein regulation (e.g., INSRTR),^101^ molecular circuit design,^103^^,^^104^ and the controlled release of therapeutic proteins from cells.^105^^,^^108^ They also serve as engineered activators of calcium channels,^110^ expanding their utility in modulating cellular functions.

CCs have immense potential in mammalian cell regulation and are expected to witness further implementations due to their small size, versatility, and tunability. The selection of CC assembly hinges on the desired activity outcome. For a robust and swift response, one would opt for the strongest CC pairs available. Conversely, for a slower, more modest response, a weaker pair could be preferable. As easily applied, non-covalent interactions, they offer highly modular solutions for regulating cellular processes of interest with a small genetic footprint. Recent advancements in synthetic biology point to a promising future for CCs, paving the way for innovative therapeutic interventions and biomedical applications. Thus, harnessing the potential of CCs represents a compelling avenue for advancing our understanding of cellular mechanisms and developing novel strategies for biosynthesis, disease management and treatment.
